# Tazemetostat decreases β-catenin and CD13 protein expression in HEPG-2 and Hepatitis B virus-transfected HEPG-2 with decreased cell viability

**DOI:** 10.1186/s13148-023-01593-8

**Published:** 2023-11-08

**Authors:** Mohamed N. Amin, Yousra M. El-Far, Mohammed El-Mowafy, Abdelaziz Elgaml

**Affiliations:** 1 Biochemistry Department, Faculty of Pharmacy, Mansoura University, Mansoura, 35516 Egypt; 2 Microbiology and Immunology Department, Faculty of Pharmacy, Mansoura University, Mansoura, 35516 Egypt; 3 Microbiology and Immunology Department, Faculty of Pharmacy, Horus University, New Damietta, 34518 Egypt

**Keywords:** Cancer stem cells, CD13, EZH2, HBV, HCC, HEPG2

## Abstract

Hepatocellular carcinoma (HCC) is one of the global health concerns. Hepatitis B virus (HBV) is one of the major causes of HCC. Poor clinical outcome of HCC patients is attributed to a small population of cancer cells known as cancer stem cells (CSCs). In this work, we studied the effect of inhibiting the enhancer of zeste homologue 2 (EZH2), a histone methyltransferase known to be overexpressed in CSCs, using tazemetostat (Taz). The effect of Taz was assessed in the HCC cell line (HEPG2) and Hepatitis B virus-transfected HEPG2 (HBV/HEPG2) cells. MTT assay showed a significant decrease in HEPG2 cells viability after 48 h treatment with either 0.5, 1, 4 or 6 μM Taz. HEPG2 and HBV/HEPG2 cells were incubated with either 0.5 or 1 μM Taz for 48 h, and then, the cells and supernatants were collected for protein expression analysis of EZH2, CD13, epithelial cell adhesion molecule (EpCAM) and β-catenin using enzyme-linked immunosorbent assay (ELISA). Taz showed a significant dose-dependent inhibition of EZH2, CD13 and β-catenin in HEPG2 and HBV/HEPG2 cells. Also, EpCAM protein levels were significantly decreased in HBV/HEPG2 but not in HEPG2 cell line alone. Our results indicate that Taz inhibition of EZH2 leads to downregulation of β-catenin signaling and eventually decreased expression of CD13 and EpCAM, which are characteristic for CSCs. The present study suggests that Taz could be a promising treatment for HCC including HBV-induced HCC that might be used in combination with radio/chemotherapy to target CSCs and prevent tumor relapse.

## Highlights

- Taz decreases the levels of EZH2, CD13 and β-catenin in HEPG2 and HBV/HEPG2 cells.
- Taz decreases the levels of EpCAM in HBV/HEPG2 but not in HEPG2 cells.
- Taz could be a promising treatment for HCC including HBV-induced HCC.

## Introduction

Hepatocellular carcinoma (HCC) is one of the worldwide-spreading malignant neoplasms, accounting for nearly 90% of all cancer cases derived from hepatocytes [1]. Cirrhosis is considered as one of the well-defined risk factors for HCC development [2, 3]. It is well reported that hepatitis B virus (HBV) is one of the most common causes of cirrhosis that eventually leads to HCC [4–6]. Despite HCC treatment gained a lot of interest, the prognosis of this disease remains elusive and new avenues and trends regarding HCC progression and tumorigenesis must be studied for better outcomes. Researchers have demonstrated that inferior clinical consequences of HCC patients are associated with certain type of cancer cells known as cancer stem cells (CSCs). They have been identified as a small set of cancer cells that can reconstitute malignancy, and therefore, they have a pivotal function during tumorigenesis and metastasis as well as resistance to chemo/radiotherapy. Thus, CSCs can lead to relapse of HCC after standard treatment regimens [7]. Hence, it is necessary to discover novel efficient drugs to target hepatic CSCs selectively.

Epithelial cell adhesion molecule (EpCAM), which is a type I transmembrane glycoprotein, is frequently expressed in multiple carcinomas and plays an important role in cell–cell adhesion process. It has been reported that EpCAM participates in cellular proliferation, metastasis as well as cellular differentiation. Evidences revealed that EpCAM is a useful marker for CSCs in HCC, not only that, but also it is found to be highly expressed in patients infected with HBV [8]. EpCAM is known to be a direct transcriptional target of Wnt/β-catenin signaling in HCC cells. This suggestion was confirmed by previous study conducted by Yamashita et al., which revealed that blocking of Wnt/β-catenin axis exerted a significant downregulation in EpCAM expression pattern [9].

CD13, a membranous glycoprotein, is reported to be closely correlated with tumor chemo-resistance/relapse following treatment with standard anticancer drugs [10]. Recent clinical study by Yamanaka et al., suggested that the high expression of CD13 in HCC patients was significantly associated with early recurrence, and hence poor prognosis of patients as well as shorter survival [11].

Wnt/β-catenin signaling transduces evolutionarily conserved pathways, which play ever-expanding role in regulation of different cellular activities, and hence normal tissue homeostasis. Evidence revealed that Wnt/β-catenin signaling axis contributes to HCC progression, as nearly one third of HCC cases were reported with its over-activation [12, 13]. Additionally, it has been demonstrated that overexpression of β-catenin is responsible for differentiation of normal hepatocytes into malignant hepatocyte progenitors and maintains CSCs self-renewal capacity. Therefore, β-catenin overexpression can eventually lead to development of cancer chemo/radio resistance and HCC recurrence [14, 15].

Enhancer of zeste homolog-2 (EZH2) is a member of the polycomb group protein family, which modifies the transcription at the epigenetic level by regulating histone and DNA methylation [16]. EZH2 is known to be included in regulation of DNA replication, cellular division and cell cycle progression. Consequently, dysregulation in EZH2 function results in tumorigenesis. EZH2 has been shown to have an oncogenic role in cancer development and progression [17]. Furthermore, extensive studies demonstrated that EZH2 could increase cancer proliferation, angiogenesis and invasion in different malignancies, including breast cancer [18], melanoma [19] and oral squamous cell carcinoma [20] among others. Moreover, EZH2 is involved in maintaining CSCs expansion and self-renewal capabilities through its action on several signaling pathways including Wnt/β-catenin and its downstream transcriptional targets such as EpCAM [21, 22].

Accumulating data have revealed that inhibition of EZH2 by either selective inhibitors or gene knockdown results in tumor inhibition [17]. Because of the reported definitive role of EZH2 dysregulation in cancer initiation and progression, previous studies have shed the light on the use of selective EZH2 inhibitors in controlling carcinogenesis along with examining their safety profile to be administered clinically to patients with different malignancies [23]. Tazemetostat (Taz) (EPZ-6438) is S-adenosylmethionine-competitive inhibitor of EZH2 with a pyridine-amide core. It has been reported that the remarkable selective inhibitory activity of Taz is mainly attributed to its binding capability to the unique split catalytic domain of polycomb repressive complex-2 (PRC2) [24]. Preliminary clinical trials for treatment of Hodgkin’s lymphoma or malignant rhabdoid tumor with Taz have been established, which were encouraging as partial and complete responses were obtained after administration of Taz to patients [25]. Therefore, we were motivated in this study to evaluate the effect of Taz as selective inhibitor of EZH2 on targeting liver CSCs via affecting Wnt/β-catenin axis using either HEPG2 cells or HBV transfected HEPG2 (HBV/HEPG2) cells.

## Materials and methods

### HEPG2 cell culture and Taz treatment

HEPG2 cell line was obtained from ATCC via Nawah-Scientific, Cairo, Egypt. Cells were maintained at Dulbecco's Modified Eagle Medium–high glucose (DMEM; #12-604F, LONZA, UK) with 10% fetal bovine serum (FBS; #S-001-BR, LSP, UK), 100 U/ml penicillin and 100 µg/ml streptomycin incubated at 37 °C and 5% CO_2_. Cells were sub-cultured when reached 90% confluency. For assessment of Tazemetostat (Taz; EPZ-6438, Adipogen, San Diego, CA) effect, HEPG2 cells were cultured in 6-well plate at cell density of 10^5^ cell/cm^2^. On the next day, medium was changed to DMEM with 2% FBS and cells were treated with either 2 nM, 0.5 or 1 μM Taz dissolved in dimethylsulfoxide (DMSO). As controls, cells in separate wells were untreated, and others were treated with equivalent concentration of DMSO (0.05% v/v). After 48 h incubation, supernatants were collected, cells were trypsinized and cell pellets were collected by centrifugation and stored at − 80 C° for further analysis.

### Transfection of HEPG2 cells with HBV and Taz treatment

HEPG2 cells were transfected by HBV plasmid 1.3-mer WT (a gift from Wang-Shick Ryu, Addgene plasmid #65459) [26] using Lipofectamine 2000 (#11668019, Thermo Fisher Scientific, USA) and Opti-MEM (#31985062, Thermo Fisher Scientific, USA). Briefly, HEPG2 cells were cultured till 70% confluency in 6-well plate as mentioned in the previous section, then the medium was aspirated and cells washed once with 1 × phosphate buffer saline (PBS). Thereafter, transfection mixture (16 μl Lipofectamine 2000, 6 μg plasmid DNA and 400 μl Opti-MEM) was added and cells were incubated at 37 °C and 5% CO_2_. Cells in separate wells received transfection mixture without HBV vector and served as vector control. After 1 h, 2 ml of Opti-MEM with 5% FBS was added to each well and cells were incubated for 48 h. At the end of incubation, supernatant was collected for analysis of HBV antigens titer (data not shown) to confirm successful transfection and Taz treatment was initiated. HBV/HEPG2 cells were incubated with either 0.5 μM or 1 μM Taz for 48 h in DMEM medium containing 2% FBS. Cells in separate wells were either Taz-untreated or received 0.05% DMSO to serve as HBV and DMSO controls, respectively. Finally, supernatants and cell pellet were collected and stored at − 80 °C for further analysis.

### ELISA assay

Cell supernatants were used for measurement of EpCAM levels using commercially available ELISA kit (#EHEPCAM, Thermo Fisher Scientific, Waltham, MA) according to the manufacturer's instructions. Cell pellet was washed 3 times with PBS, then resuspended in PBS. Cell lysis was achieved by 3 cycles of freezing to − 20 °C and thawing at room temperature. Cell lysate was centrifuged at 1500×*g* for 10 min at 4 °C to remove cellular debris, and then, supernatant was collected for further analysis. Protein expression levels of EZH2, CD13 and β-catenin were assessed using commercially available ELISA kits (#LS-F7278, Seattle, USA; #ab239427, Abcam, Cambridge, UK; #LS-F20972 Seattle, USA, respectively) according to the manufacturer's instructions. Absorbance was measured using microplate reader (ELx800, BioTek Instruments, USA) and BioTek’s Gen5™ Data Analysis Software.

### MTT cell viability assay

HEPG2 cells were seeded in 96-well plate with cell density of 10^4^ cells/well. After 24 h incubation, medium was changed to DMEM with 2% FBS and Taz was added in either 1, 2 nM, 0.5, 1, 4 or 6 μM concentration in separate wells. Cells treated with DMSO at final concentration of 0.05% were considered as the baseline cell viability. Cells were incubated for 48 h at 37 °C and 5% CO_2_. At the end of incubation period, cells were subjected to MTT cell viability assay using 3-(4,5-dimethylthiazol-2-yl)-2,5-diphenyl tetrazolium bromide (#20,395.01, SERVA, Heidelberg, Germany). Briefly, medium was aspirated, cells were washed with 1 × PBS and treated with 20 µl MTT solution (5 mg/ml) for 4 h incubation period at 37 °C. Then, 100 µl DMSO were added into each well. The optical density of each well was measured at 570 nm using a microplate reader (ELx800, BioTek Instruments, USA) and BioTek’s Gen5™ Data Analysis Software. The mean cell viability percentage was calculated relative to control wells of cells treated with DMSO alone.

### Statistical analysis

All experiments were performed in triplicates. Data were represented as mean ± SEM. Means were compared using one-way ANOVA with Tukey post hoc test. Statistical analysis was done using IBM SPSS Statistics for Windows, Version 25.0. Armonk, NY: IBM Corp.

## Results

### Taz decreased the viability of HEPG2 cells

**Fig. 1:**
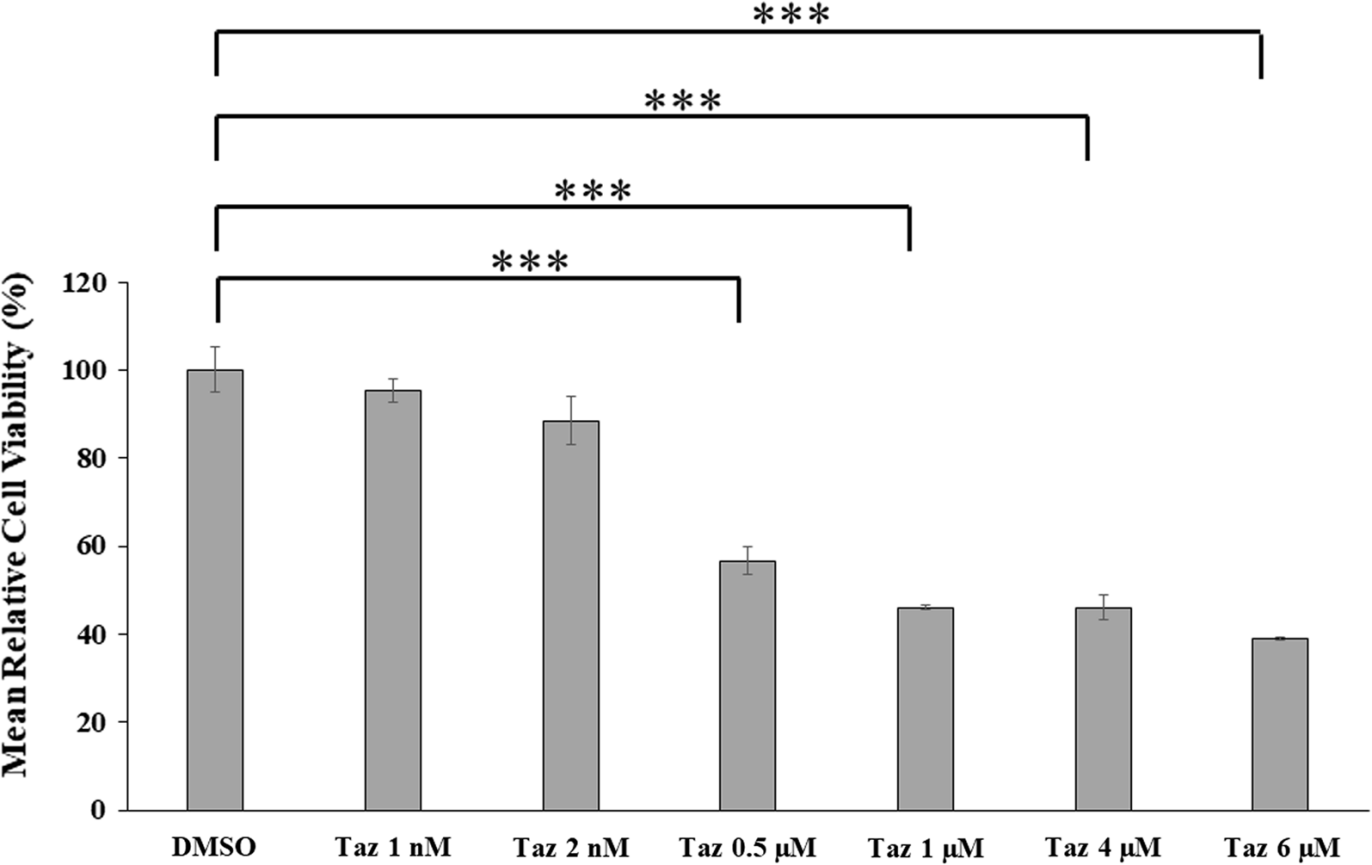
MTT cell viability assay of HEPG2 cells after 48 h incubation with 1 nM, 2 nM, 0.5 μM, 1 μM, 4 μM and 6 μM concentrations of Taz versus DMSO control; (***): *P* < 0.001

**Fig. 2:**
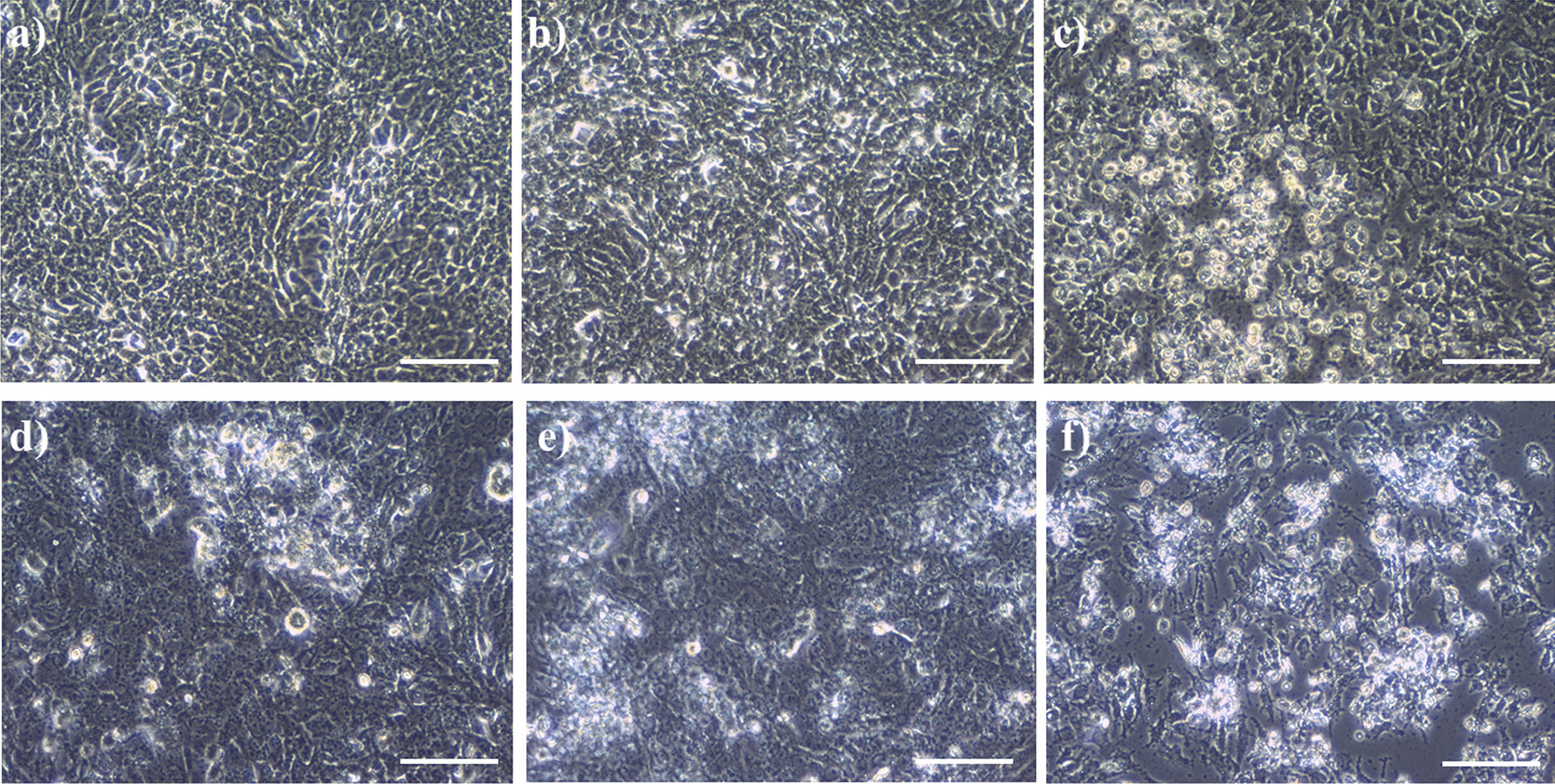
Phase contrast images of different cell groups after 48 h of different treatments. **a** untreated HEPG2 cells; **b** HEPG2 with 0.05% DMSO; **c** HEPG2 with 1 μM Taz; d) HEPG2 transfected with hepatitis B virus (HBV/HEPG2); **e** HBV/HEPG2 with 0.05% DMSO; **f** HBV/HEPG2 with 1 μM Taz; Scale Bar: 100 µm

Taz decreased the mean relative cell viability (cell viability) for HEPG2 cells nearly in a consistent manner with increasing Taz concentration. Taz concentrations of 1 nM and 2 nM decreased HEPG2 cell viability with no significant difference compared to DMSO control (Fig. 1). In addition, increasing Taz concentration to either 0.5, 1, 4 or 6 μM significantly (*P* < 0.001) decreased HEPG2 cell viability compared to DMSO control (Fig. 1). Such results agree with phase contrast images of HEPG2 cells after 48 h incubation with 1 μM Taz (Fig. 2C) depicting obvious decreased cell viability, faint cellular appearance and decreased cellular attachment to culture surface compared to DMSO control (Fig. 2B). Untreated HEPG2 cells are depicted in (Fig. 2A).

### Taz decreased EZH2 expression in HEPG2 and HBV/HEPG2 cells in a dose-dependent manner

**Fig. 3:**
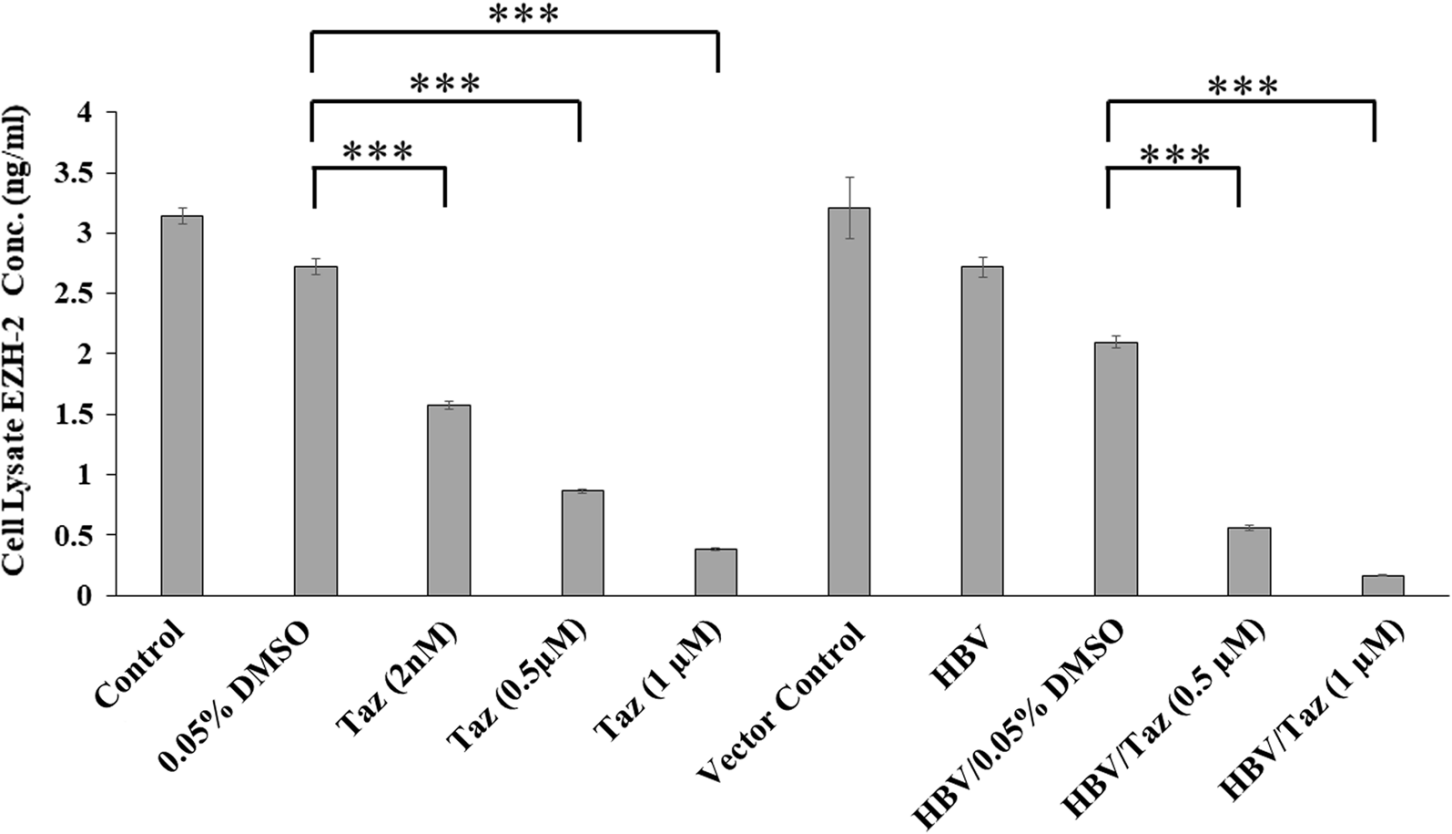
EZH-2 protein expression levels (ng/ml) in cell lysate of HEPG2 cells or HEPG2 transfected with hepatitis B virus (HBV/HEPG2) after 48 h incubation with different concentrations of Taz or 0.05% DMSO; (***): *P* < 0.001

EZH2 protein levels significantly decreased in HEPG2 incubated with 2 nM, 0.5 μM and 1 μM Taz compared to DMSO control (*P* < 0.001) (Fig. 3). Similarly, in HBV/HEPG2 EZH2 protein levels were significantly lower when treated with 0.5 and 1 μM Taz compared to DMSO control (*P* < 0.001) (Fig. 3).

### Taz decreased β-catenin expression in HEPG2 and HBV/HEPG2 cells in a dose-dependent manner

**Fig. 4:**
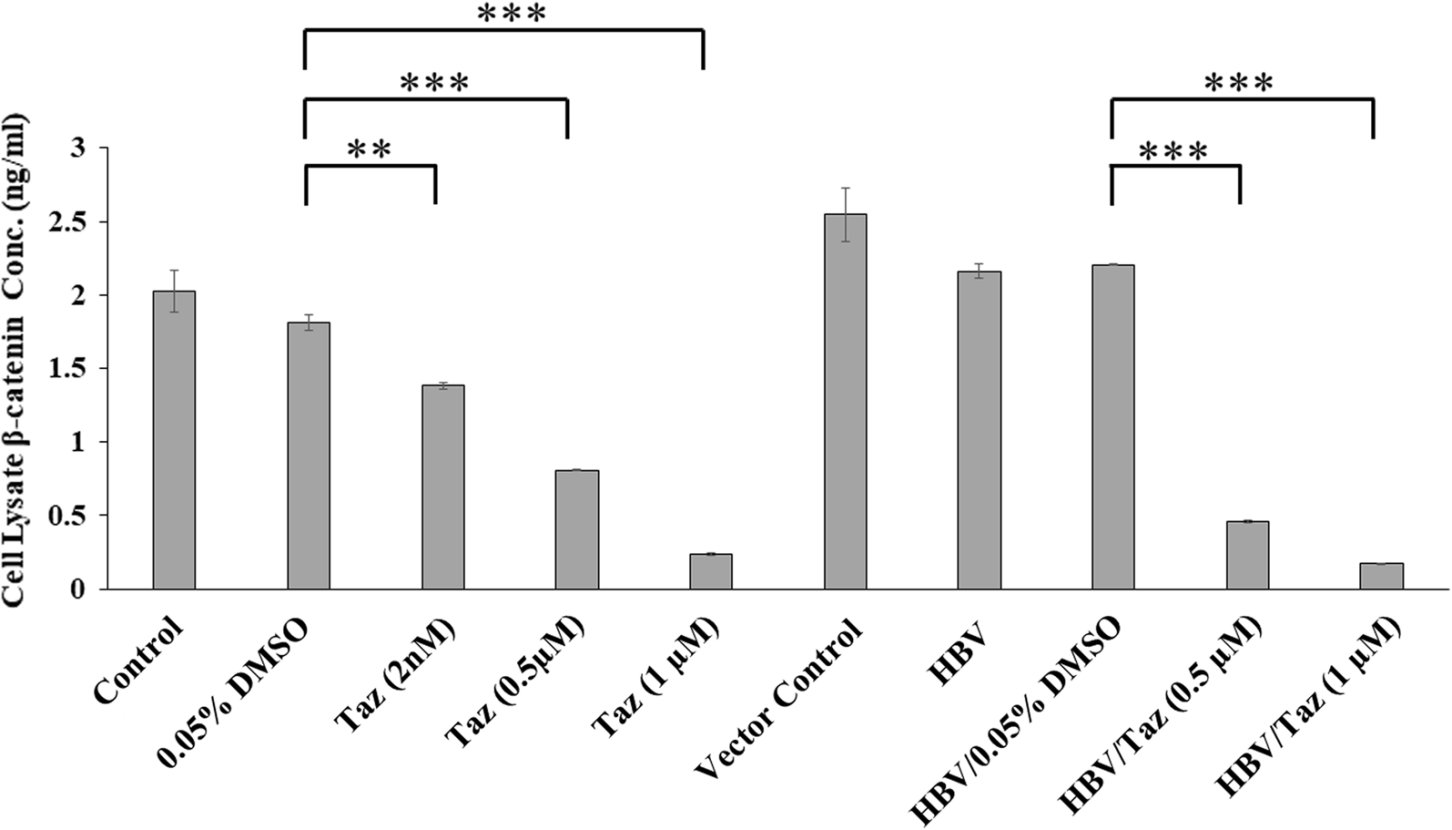
β-catenin protein expression levels (ng/ml) in cell lysate of HEPG2 cells or HEPG2 transfected with hepatitis B virus (HBV/HEPG2) after 48 h incubation with different concentrations of Taz or 0.05% DMSO; (**): *P* < 0.01; (***): *P* < 0.001

The protein expression of β-catenin significantly decreased in HEPG2 treated with 2 nM Taz compared to DMSO control group (*P* < 0.01) (Fig. 4). Also, HEPG2 treated with 0.5 μM and 1 μM Taz showed a significant lower expression of β-catenin (*P* < 0.001). As for HBV/HEPG2, β-catenin levels significantly (*P* < 0.001) decreased when treated with 0.5 and 1 μM Taz, respectively, as compared to their counterpart DMSO control (Fig. 4).

### Taz decreased CD13 expression in HEPG2 and HBV/HEPG2 cells in a dose-dependent manner

**Fig. 5:**
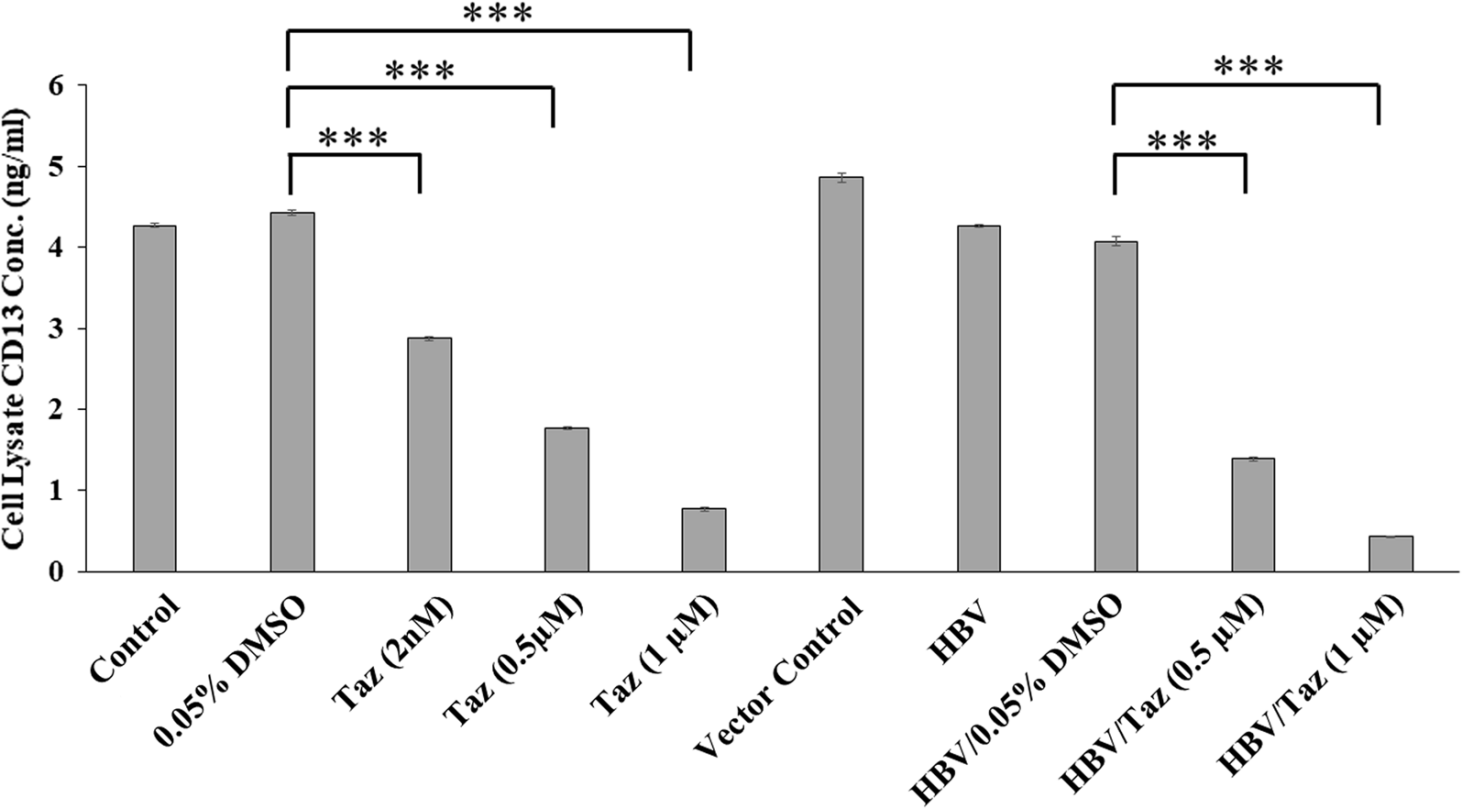
CD13 protein expression levels (ng/ml) in cell lysate of HEPG2 cells or HEPG2 transfected with hepatitis B virus (HBV/HEPG2) after 48 h incubation with different concentrations of Taz or 0.05% DMSO; (***): *P* < 0.001

Treatment of HEPG2 with either 2 nM, 0.5 or 1 μM Taz significantly decreased CD13 protein levels compared to DMSO control (*P* < 0.001) (Fig. 5). Moreover, HBV/HEPG2 showed a similar pattern with either 0.5 μM or 1 μM Taz significantly decreasing CD13 levels compared to their counterpart DMSO control. Treating HEPG2 or HBV/HEPG2 with 1 μM Taz decreased CD13 levels to as low as 0.77 ± 0.02 and 0.43 ± 0.002 (ng/ml), respectively.

### Taz significantly decreased EpCAM expression in HBV/HEPG2 cells

**Fig. 6:**
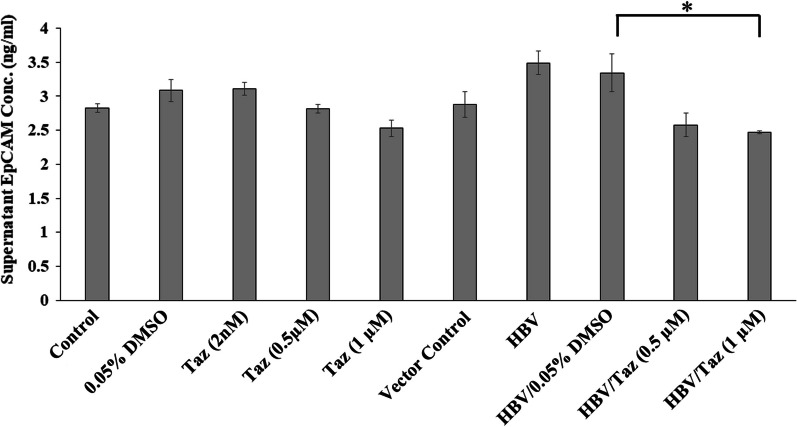
EpCAM protein expression levels (ng/ml) in the supernatant of HEPG2 cells or HEPG2 transfected with hepatitis B virus (HBV/HEPG2) after 48 h incubation with different concentrations of Taz or 0.05% DMSO; (*): *P* < 0.05

HEPG2 cells treated with 2 nM Taz showed nearly the same EpCAM protein expression levels as DMSO control (Fig. 6). In addition, treating HEPG2 with 0.5 μM or 1 μM Taz decreased EpCAM levels to 2.81 ± 0.06 and 2.52 ± 0.11 (ng/ml), respectively, compared to 3.08 ± 0.16 (ng/ml) in DMSO control. However, still there was no significant difference. As expected, HBV transfection increased EpCAM expression in HBV/HEPG2 compared to the vector control. On the other hand, treating HEPG2 with 0.5 or 1 μM Taz decreased EpCAM levels to 2.58 ± 0.16 and 2.46 ± 0.02 (ng/ml), respectively, compared to 3.34 ± 0.27 (ng/ml) in their HBV/DMSO control. The difference was statistically significant in case of HBV/HEPG2 treated with 1 μM Taz (*P* < 0.05). The results were consistent with phase contrast images of HBV/HEPG2 cells treated with 1 μM Taz (Fig. 2F) showing floating dead cells and decreased cell count compared to its counterpart DMSO control cells (Fig. 2E). Untreated HBV/HEPG2 cells are illustrated in (Fig. 2D)

## Discussion

EZH2 is a histone methyltransferase and is considered as a catalytic subunit of initiation complex PRC2 that regulates gene expression through catalyzing tri-methylation of histone H3 at lysine 27 residue [27]. Accumulating evidence from in vitro and in vivo models together with clinical studies collectively suggests that EZH2 is upregulated in various malignant solid tumors such as lung, hepatocellular, ovarian, colorectal and breast cancers. It has been well-established that EZH2 promotes cell survival, proliferation, epithelial-mesenchymal transition, invasion and drug resistance of cancer cells through downregulation of tumor suppressor genes expression and oncogenes upregulation [28]. More importantly, EZH2 has been found to be overexpressed in CSCs of various malignancies and possess an important function in expansion, maintenance and pluripotency of CSCs through its action on several signaling pathways; most importantly Wnt/β-catenin signaling pathway [22, 29].

Nowadays, epigenetic targets represent promising new avenues for cancer therapy development. In the current study, we studied the in vitro antitumor activity of the selective S-adenosylmethionine-competitive inhibitor of EZH2 known as tazemetostat (Taz) using HEPG2 cells and HBV/HEPG2 cells. Taz is a potent and highly specific EZH2 inhibitor [30, 31]. Importantly, it has been recently approved as safe and well tolerated drug by United States Food and Drug Administration (USFDA). It is the first epigenetic drug approved for administration to patients suffering from solid tumors. Furthermore, Taz exerted a beneficial clinical importance in group of patients with advanced epithelioid sarcoma [32]. Our results showed that Taz exerted a significant inhibition of EZH2 protein expression in HEPG2 and HBV/HEPG2 cells in a dose-dependent manner as compared to control groups.

EpCAM is a pan-epithelial differentiation carcinoma-associated antigen that has been reported to be up-regulated in rapidly proliferating tumor cells in most human epithelial carcinomas. EpCAM serves as a biomarker of HCC. Also, it has been reported that EpCAM^+^ HCC cells commonly possess CSCs like properties including the capacity for self-renewal, differentiation, tumorigenesis and a signature of chemotherapy resistance [7, 33, 34].

Data from our in vitro study demonstrated that Taz at different concentrations showed a significant reduction in β-catenin protein expression in HEPG2 cell lysate as compared to the corresponding DMSO control group. Taz at 0.5 or 1 μM concentrations exerted a more pronounced decrease in β-catenin protein expression as compared to 2 nM concentration. These data indicate that Taz as a potent and specific inhibitor of EZH2 could significantly decrease EZH2 protein expression along with significant reduction in β-catenin protein expression in a dose-dependent manner. It has been demonstrated that EpCAM is a downstream transcriptional target of Wnt/β-catenin pathway in HCC cells. Moreover, it plays an important role in the activation of Wnt signaling [35]. EpCAM and Wnt/β-catenin pathway are functionally connected and share a significant role in tumor progression and increasing the stemness properties of hepatic CSCs [9, 36].

Human HCC progression and development is maintained by CSCs expressing CD13. The advanced disease stage, larger tumors and poor prognosis usually are correlated with increased expression of CD13 in HCC patients [36]. Data from a previous in vivo study using xenograft model for liver cancer showed that CD13 inhibition had a potent suppressive effect on CSCs self-renewal and tumor-initiating capabilities [10]. Our results showed that incubating HEPG2 cells with Taz, especially at 1 μM concentration, for 48 h induced a significant decrease in CD13 level. This was coupled with a non-significant decrease in EpCAM protein concentration by 0.17 fold indicating that it might be beneficial to increase incubation time to 72 h. The observed significant decrease in cell viability after treating cells with Taz at 1 μM concentration could be attributed to its capability to target CSCs via inhibition of EZH2/β-catenin axis. These findings offer a promising therapeutic option to eradicate CSCs, and hence overcome radio/chemotherapy resistance upon using commonly standard anticancer regimens in treating HCC.

Chronic HBV infection is a major risk factor for HCC development. It is well documented that HBV X protein (HBx) acts as cofactor in HCC development and progression. It is supposed that EpCAM expression in CSCs of HBV-mediated HCC could be induced by HBx protein via active DNA demethylation directed by RelA in complex with EZH2 and TET2 in vivo [37]. Kimura et al., demonstrated that HBV is known to develop HCC faster than other etiologies including hepatitis C virus and non-B, non-C hepatitis viruses. Furthermore, HCC cells resected from most of HBV patients expressed EpCAM as stem cell marker. They also suggested that CSCs are highly expressed in HBV infected cells and have a potential toward the anticancer drug resistance. Our data showed that HBV transfection exerted an upregulation in EpCAM protein expression in HBV/HEPG2 cells as compared to corresponding vector control. These data are in accordance with CSCs theory, which suggested that CSCs are more easily formed in patients with HBV as compared to others suffering from other etiologies [8].

Collectively, we designed our study to evaluate the in vitro antitumor activity of Taz using HEPG2 cells transfected with HBV and unraveled the supposed mechanism. Treating HBV/HEPG2 with Taz at 0.5 and 1 µM concentrations exerted a significant downregulation in EZH2 protein concentration with a significant consistent decrease in β-catenin and CD13 expression levels in a dose-dependent manner. Surprisingly, incubating transfected cells with Taz at 1 µM concentration for 48 h showed a significant decrease in EpCAM expression levels as compared to the corresponding control group.

Our in vitro study revealed several new insights regarding the mechanism of Taz as a promising safe and well tolerated anticancer against HBV mediated HCC. Taz showed an inhibitory activity against hepatic CSCs via inhibiting EZH2 and consequently downregulating β-catenin, CD13 and EpCAM downstream targets. Accumulating evidence reported that EpCAM^+^ CSCs could be promoted by HBx protein through activation of β-catenin [38, 39]. Moreover, Chisari and Ferrari suggested that hepatic CSCs could be generated from hepatic progenitor cells [40].

## Conclusion

The present study clarified the inhibitory effect of Taz on EZH2, which results in alteration of β-catenin signaling, and finally diminished expression of both CD13 and EpCAM that are distinctive for CSCs. Therefore, the current work proposes that Taz can be used as a novel therapeutic approach in combination with existing radio/chemo regimens for HCC involving HBV-induced HCC to target CSCs and avoid tumor reversion. However, more future in vivo studies might be needed to confirm its clinical applicability.

## Data Availability

The data used to support the findings of this study are available from the corresponding author upon request.

## Abbreviations

CSCs: Cancer stem cells
Conc.: Concentration
EpCAM: Epithelial cell adhesion molecule
DMSO: Dimethylsulfoxide
ELISA: Enzyme-linked immunosorbent assay
EZH2: Enhancer of zeste homologue 2
HBV: Hepatitis B virus
HCC: Hepatocellular carcinoma
HEPG2: Human hepatocellular carcinoma cell line
HBV/HEPG2: HEPG2 cell line transfected with hepatitis B virus plasmid
PBS: Phosphate buffer saline
PRC2: Polycomb repressive complex-2
Taz: Tazemetostat
